# Update on LIPID MAPS classification, nomenclature, and shorthand notation for MS-derived lipid structures

**DOI:** 10.1194/jlr.S120001025

**Published:** 2020-10-09

**Authors:** Gerhard Liebisch, Eoin Fahy, Junken Aoki, Edward A. Dennis, Thierry Durand, Christer S. Ejsing, Maria Fedorova, Ivo Feussner, William J. Griffiths, Harald Köfeler, Alfred H. Merrill, Robert C. Murphy, Valerie B. O’Donnell, Olga Oskolkova, Shankar Subramaniam, Michael J. O. Wakelam, Friedrich Spener

**Affiliations:** 1 Institute of Clinical Chemistry and Laboratory Medicine, Regensburg University Hospital, Regensburg, Germany; 2 Department of Bioengineering, University of California San Diego, La Jolla, CA, USA; 3 Graduate School of Pharmaceutical Sciences, University of Tokyo, Bunkyo-ku, Tokyo, Japan; 4 Department of Chemistry and Biochemistry, Department of Pharmacology, School of Medicine, University of California San Diego, La Jolla, CA, USA; 5 Institute of Biomolecules Max Mousseron, University of Montpellier, CNRS, ENSCM, Montpellier, France; 6 Department of Biochemistry and Molecular Biology, Villum Center for Bioanalytical Sciences, University of Southern Denmark, Odense, Denmark; 7 Cell Biology and Biophysics Unit, European Molecular Biology Laboratory, Heidelberg, Germany; 8 Institute of Bioanalytical Chemistry, Faculty of Chemistry and Mineralogy, University of Leipzig, Leipzig, Germany; 9 Center for Biotechnology and Biomedicine, University of Leipzig, Leipzig, Germany; 10 Department of Plant Biochemistry, Albrecht-von-Haller-Institute and Göttingen Center for Molecular Biosciences (GZMB), University of Göttingen, Göttingen, Germany; 11 Swansea University Medical School, Swansea, United Kingdom; 12 Core Facility Mass Spectrometry, Medical University of Graz, Graz, Austria; 13 School of Biological Sciences and the Parker H. Petit Institute for Bioengineering and Bioscience, Georgia Institute of Technology, Atlanta, GA, USA; 14 Department of Pharmacology, University of Colorado at Denver, Aurora, CO, USA; 15 Systems Immunity Institute, Cardiff University, Cardiff, United Kingdom; 16 Institute of Pharmaceutical Sciences, University of Graz, Graz, Austria; 17 Department of Biomedical Engineering, Jacobs School of Engineering, University of California San Diego, La Jolla, CA, USA; 18 Babraham Institute, Babraham Research Campus, Cambridge, United Kingdom; 19 Department of Molecular Biosciences, University of Graz, Graz, Austria; 20 Division of Molecular Biology and Biochemistry, Gottfried Schatz Research Center, Medical University of Graz, Graz, Austria

**Keywords:** mass spectrometry, lipidomics, fatty acyls, glycerolipids, glycerophospholipids, sphingolipids, sterols

## Abstract

A comprehensive and standardized system to report lipid structures analyzed by MS is essential for the communication and storage of lipidomics data. Herein, an update on both the LIPID MAPS classification system and shorthand notation of lipid structures is presented for lipid categories Fatty Acyls (FA), Glycerolipids (GL), Glycerophospholipids (GP), Sphingolipids (SP), and Sterols (ST). With its major changes, i.e., annotation of ring double bond equivalents and number of oxygens, the updated shorthand notation facilitates reporting of newly delineated oxygenated lipid species as well. For standardized reporting in lipidomics, the hierarchical architecture of shorthand notation reflects the diverse structural resolution powers provided by mass spectrometric assays. Moreover, shorthand notation is expanded beyond mammalian phyla to lipids from plant and yeast phyla. Finally, annotation of atoms is included for the use of stable isotope-labeled compounds in metabolic labeling experiments or as internal standards. This update on lipid classification, nomenclature, and shorthand annotation for lipid mass spectra is considered a standard for lipid data presentation.

Lipids have become increasingly recognized as the central metabolites affecting human physiology and pathophysiology, and LIPID MAPS has recently expanded its tools, resources, data, and training as a free resource dedicated to serving the lipid research community (1). Following development of the LIPID MAPS nomenclature, classification, and structural representation system (2, 3), an initial shorthand nomenclature was proposed (4), which included a structural hierarchy as shown by others as well (5, 6). These were the first attempts to provide rules for reporting mass spectrometric data dependent on the power for structural resolution of lipids by the instrumental set-ups in use at that time.

Today, we recognize that the field has evolved in often diverging ways and that this has not enabled a unifying naming convention to be adopted throughout. For example, alternative shorthand notation has evolved for some lipid classes, a plethora of newly determined structures for lipids from various classes and phylogenetic kingdoms (higher plants and yeasts) have been described, and progress in the technological development of mass spectrometers with greater structural resolution as well as advances in automation in interpreting high-throughput data has occurred. To address this, it is the aim of this report to take into account these developments and to present an update on the LIPID MAPS classification and a pragmatic highly usable shorthand notation for those active in lipid research. This update will focus on five of the eight LIPID MAPS categories (2), namely Fatty Acyls (FA), Glycerolipids (GL), Glycerophospholipids (GP), Sphingolipids (SP), and Sterols (ST). Annotation is modified to permit annotation of oxygenated lipids and examples will be given for lipid classes occurring outside the mammalian kingdom.

“Biological intelligence” has been considered as topical knowledge about a lipid molecule, such as its structural building blocks, enzymatic pathways for generation and metabolism, and biological functions (4). Interpretation by biological evidence in shorthand notation can be useful when mass spectra contain structural ambiguities or lack of clear structural evidence. Consequently, annotations with the help of biological evidence contain assumptions, and it must be recognized and recorded that this may lead to misinterpretations. Moreover, in the pragmatic approach presented in this work, we will make more use of common and/or trivial names for the shorthand notation. For example, the structures of sterols, prostaglandins, resolvins, etc. have been characterized by chemical and spectroscopic methods, including stereochemistry, and common names exist, as do shorthand notations in many cases. Their mass spectra are also known; however, their stereochemistry and isomerism and other structural information often cannot be deduced directly from the spectra when these lipids are measured in biological samples. Assignment of a common name or of shorthand notation to such chromatographic and MS/MS data is permissible, but it may be based on annotation that includes biological intelligence, and that needs to be clearly stated as well.

In any case, assumptions made should be striking a unique balance between *what we think we know about structure and function of a lipid molecule* and what *a specific MS-based analytical method definitively informs us about the lipid structure*.

## UPDATE ON NOMENCLATURE AND CLASSIFICATION

Modification of Fatty Acyls by oxygen, either catalyzed enzymatically or by means of radical chemistry, is an important focus in biomedical research, due to the impressive biological activities of products thus obtained. Based on these two mechanisms, all compounds originating from polyunsaturated fatty acyls (PUFAs) having methylene-interrupted *cis-*double bonds (DBs) (also chemically referred to as allylic DBs) and being enzymatically or nonenzymatically oxygenated are grouped within the appropriate class in the Fatty Acyl category. Historically, the term “eicosanoid” has included “related oxygenated polyunsaturated fatty acids” with shorter or longer chain lengths, but in the LIPID MAPS classification, compounds are strictly assigned to a class based on their chain length (e.g., octadecanoids, eicosanoids, docosanoids). Recently, the common name “oxylipins”, standing for “oxygenated fatty acyls”, has come into widespread use. Similarly, in the Glycerophospholipids (GP) category, many newly described phospholipids contain oxygenated fatty acyls (or oxylipins) often termed “oxygenated phospholipids” (OxPLs). Those are produced by oxygenation of constituent fatty acyls enzymatically and nonenzymatically, or by chemical modification of polar head groups containing an amino function (PE and PS), i.e., *N*-modified phospholipids.

In the following, we elaborate first on experimental prerequisites for correct annotation of lipid mass spectrometric data and, second, present the updates on rules for using shorthand notation. Finally, in order of categories, we present mostly in the form of easily readable tables, all updates on lipid nomenclature and classification including respective shorthand abbreviations according to the LIPID MAPS web resources and the updated shorthand notation for lipid species and lipid molecular species. To further enhance the understanding of shorthand notation, some chemical structures are presented in the tables. The updated shorthand notation schemes described herein have been incorporated into a number of key resources on the LIPID MAPS website, notably the LIPID MAPS Structure Database (LMSD) and the MS search tools (see the Hierarchical concept and application of shorthand notation section below), by generating level-specific abbreviations (e.g., sum-composition and chain-specific annotations) for lipid structures. This approach is important in terms of the development of MS search databases that are appropriate for the technique used (sum-composition databases for precursor ion data and chain-composition databases for MS/MS data).

## EXPERIMENTAL PREREQUISITES FOR CORRECT ANNOTATION

All lipid species and lipid molecular species data presented need information on levels of structural resolution attained by mass spectrometric analysis, and sufficient supplementary data to justify annotation by shorthand notation. At minimum, such data should contain the measured intact *m/z* value, the adduct ion used for identification, the retention time when chromatography is applied, and the measured fragment *m/z* values.

Assignment and therefore use of specific shorthand nomenclature for defined **functional groups** (**Table 1 A–C**) requires additional techniques. An example is derivatization of hydroxyl groups by trimethylsilylation followed by GC/MS EI and analysis of fragment ions formed. In many cases ESI-MS/MS of underivatized constituent fatty acyls in general leads to specific product ions, if ESI populates a charge site near the functional group (7). Definition of DB positions can be determined by several techniques including ozonolysis during analysis (OzID) (8) or specific adduct formation with acetone in photochemical Paterno-Büchi reaction (9). These reactions can be carried out in shotgun or LC-MS/MS experiments. High energy MS/MS has been used to assign DB position of fairly complex fatty acyls as well as methyl branching (10). Alternatively, GC/MS can be used including specific derivatization of the carboxylate group, to drive specific DB fragmentation in EI spectra (11). Chemical ionization techniques are also useful by application of specific chemical ionization reagent gases to define DB positions (12).

**TABLE 1A:** Abbreviations of functional groups/side chains

Functional Group/Side Chain	Abbreviation
Ethyl branch	Et
Methyl branch	Me
Bromo	Br
Chloro	Cl
Fluoro	F
Iodo	I
Nitro	NO2
Epoxy	Ep
Peroxy	OO
Methoxy	OMe
Alkoxy (ether)	oxy
Amino	NH2
Hydroperoxy	OOH
Sulfanyl	SH
hydroxy	OH
Oxo (keto/aldehyde; depending on position)	oxo
Cyano	CN
Phosphate	P
Sulfate	S
Carboxylic acid	COOH
Glycine	G
Taurine	T

The order of functional groups aligns with IUPAC hierarchy (14).

**TABLE 1B:** Abbreviations of cyclic structures

Cyclic Structures	Abbreviation
Cyclopropyl	cy3
Cyclopropenyl	cy3:1
Cyclobutyl	cy4
Cyclopentyl	cy5
Cyclohexyl	cy6

**TABLE 1C:** Abbreviations of carbohydrate structures

Carbohydrate Structures	Abbreviation
Hexose	Hex
Galactose	Gal
Glucose	Glc
Mannose	Man
Neuraminic acid	Neu
*N*-acetyl hexosamine	HexNAc
*N*-acetyl galactosamine	GalNAc
*N*-acetyl glucosamine	GlcNAc
*N*-acetyl neuraminic acid	NeuAc
*N*-glycolylneuraminic acid	NeuGc
Keto-deoxy-glycero-galacto-nononic acid	Kdn
Glucuronic acid	GlcA
Xylose	Xyl
Fucose	Fuc

Glycan annotation is based on IUPAC-approved abbreviations (https://www.ncbi.nlm.nih.gov/glycans/snfg.html) (15).

Common names of lipid species, e.g., for certain fatty acids and for oxygenated fatty acids denote a chemically defined structure including stereochemistry. For proper annotations in these cases, the analytical method has to provide for chiral separation of known stereoisomeric compounds. This validation demands data on reproducibility and limit of quantification. Similarly, when novel structures are described, analytical details proving structural details need to accompany the data. Guidelines for method validation and reporting of novel lipid molecules are currently being developed within the Lipidomics Standard Initiative (https://lipidomics-standards-initiative.org) as community-wide effort (13).

## UPDATES ON GENERAL RULES FOR SHORTHAND NOTATION

Here, we describe updates and rules applicable to all lipid categories described below. This includes rules on the hierarchical concept and application of the nomenclature and annotation of lipid structures as well as on annotation of stable isotope-labeled lipids. Three major updates are:

- The term “DBs” is replaced by “double bond equivalents” (DBEs), because removal of two hydrogen atoms from precursor lipid forms a double bond, an oxo group or a cyclic structure. Frequently, MS does not distinguish between these alternatives.
- Oxygen atoms represent not only the main component introduced during oxygenation, but occurs also in hydroxy groups as a principal structural feature in many lipid classes such as sphingoid bases. Because hydroxy, oxo or other oxygen functional groups may not be differentiated by high resolution/accurate mass analysis, annotation is done by the number of oxygens linked to the hydrocarbon chain.
- Use of parentheses and brackets is minimized. Parentheses indicate primarily positions and, with regard to functional groups only those with numbers behind them, like (OH)2, (NO2), (NH2). The use of square brackets is restricted to chemical configurations R and S, to stable isotopes, and to the frame of carbons in a ring structure.

### Hierarchical concept and application of shorthand notation

- Upon application of a validated MS-method, interpretation of mass spectra by “biological intelligence” and the use of common or trivial names, as alluded to specifically in the introduction, is permissible. Such annotations need to be clearly stated. Examples are ambiguities pertaining to bond type, oxygenated groups, and branched chains.
- “Species level” is now the lowest hierarchical level. It represents the sum composition, i.e., sum of carbon atoms, DBEs, and number of additional oxygen atoms, e.g., FA 18:1;O. It thus replaces former “Lipid class level” mass (i.e., lipid class and the – uncharged - molecular mass). Of note, for sterols, the ABCD ring system is assumed and not expressed as DBE.
- “Phosphate-position level” annotates positions of phosphate group(s), e.g., PIP(3′) or PIP2(4′,5′) at phosphatidylinositolphosphate.
- “Molecular species level” pertains to all categories addressed here and is reached as soon as constituent fatty acyl/alkyl-residues are identified, e.g., TG 16:0_18:1_18:1, a triglyceride.
- “*sn*-position level” is a more refined level in GL and GP categories, enabling annotation of the *sn*-position of fatty acyl/alkyl constituents at the glycerol backbone as indicated by a slash, e.g., TG 16:0/18:1/18:1.
- “DB-position level” or “DBE-position level” pertain to species having constituents with defined position of double bonds or double bond equivalents, e.g., FA 18:2 (9, 11);O.
- “Structure defined level” annotates molecular species composed of various constituents and functional groups, yet without positions and stereochemical details, e.g., FA 18:2;OH.
- “Full structure level” annotates molecular species composed of various constituents and functional groups including positions, yet without stereochemical details, e.g., FA 18:2(9Z,11E);13OH.
- “Complete structure level” defines detailed structures of all functional groups including stereochemistry as shown in the LMSD, e.g., 13R-HODE, 13S-HODE (= common name).

**Fig. 1.**
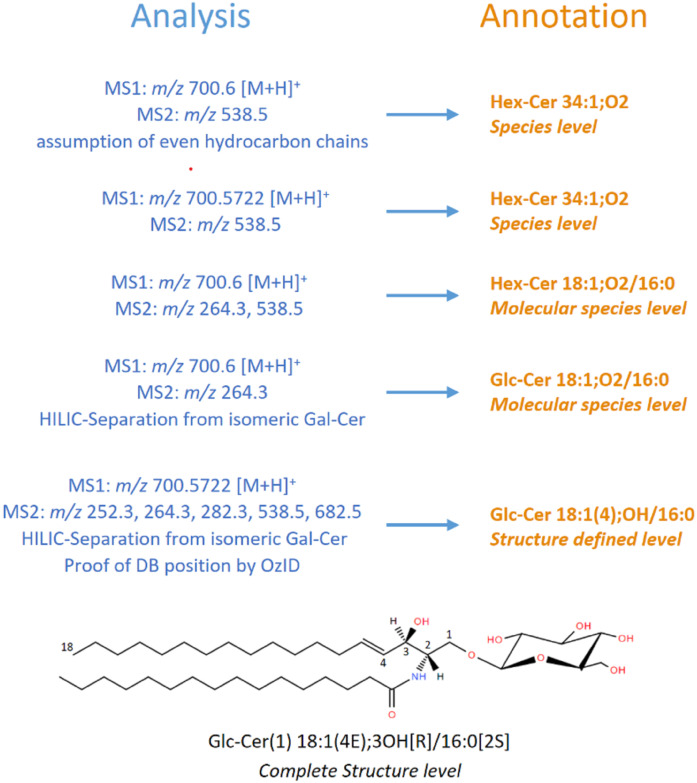
Hierarchical scheme for the analysis of glucosylceramide. “Analysis” presents MS-data and “Annotation” the respective hierarchical *levels* with corresponding shorthand annotation. The chemical structure illustrates the *Complete Structure level*, numbers along the sphingoid base indicate conventional numbering of carbons therein.

**Figure 1** presents such a hierarchical scheme, taking the example of glucosylceramide.

A word of caution is appropriate here: Annotations based solely on *m/z* features and on returns from database retrieval are frequently incorrect due to over-interpretation of experimental data, i.e., returns of chemically defined lipid molecules at Complete structure level. It is therefore of major importance that database search tools return appropriate annotations based on sum composition, i.e., at Species level and Molecular species level. Such tools are, for example, the LIPID MAPS MS search tools (https://lipidmaps.org/resources/tools/bulk_structure_searches_overview.php) (see also comment in the discussion) or the “ALEX lipid calculator” (http://alex123.info/ALEX123/MS.php).

### Annotation of lipid structures

- Lipid species are annotated by class shorthand abbreviation (see Tables 2A-6A), followed by a space and C-atoms:DBE, e.g., TG 54:5, or C-atoms:DBE;O-atoms in fatty acyl/alkyl residues, e.g., FA 18:1;O or PC 38:3;O2.
- Variable constituents like fatty acyls/alkyls are assigned based on their mass as number of C-atoms and number of DBE (C-atoms:DBE), when experimental proof for DB is provided the annotation is C-atoms:DB. Where applicable, the number of oxygen-atoms is added, separated by a semi-colon, e.g., C-atoms:DBE;O-atoms.
- DB-position is indicated by a number according to D -nomenclature (geometry unknown) or a number followed by geometry (Z for *cis*, E for *trans*). Specific techniques are required for determination of DB-position (or geometry) to validly use this level of annotation, e.g., FA 18:2 (9, 12), FA 18:2(9Z,12Z).
- Positions for all functional groups are stated in front of functional group abbreviation, e.g., FA 20:4;12OH.
- Generally, all functional groups (see for abbreviations) are separated by a **semicolon** after the number of DBE. Functional groups are placed **inside a separate pair of parentheses, only** if more than one followed by the number of groups, e.g., FA 20:3;(OH)2;oxo. Moreover, functional groups containing numbers such as NO2 or NH2 are generally placed inside a separate pair of parentheses, e.g., FA 18:1;(NO2). The order of functional groups follows the IUPAC hierarchy (14).
- Except for DBE/DB-position, **proven** positions of all other functional groups are stated according to D -nomenclature in front of the functional group abbreviation that are separated by a comma if more than one, e.g., FA 20:3(5Z,13E);11OH,15OH;9oxo
- Cyclic structures cyX (X = number of ring atoms, see for abbreviations) are presented in front of other functional groups. Their structural details are annotated within a pair of square brackets. Within the square brackets the positions of ring atoms, separated by hyphen, are placed in front of the cyX annotation. Other functional groups are placed after the ring structure of the cyX annotation, e.g., FA 20:2;[8-12cy5;11OH;9oxo];15OH = 8-iso-PGE_2_ or PGE_2_.
- Carbohydrate structures (), e.g., in complex glycosphingolipids, are annotated as described for glycans (https://www.ncbi.nlm.nih.gov/glycans) (15). When the sequence of sugars components is known they are shown in this order separated by a hyphen, e.g., Gal-Glc-Cer 18:1;O2/16:0. In case the sequence is unknown the components (followed by their number if more than one) are shown in alphabetic order in front of the respective lipid backbone, e.g., Gal2GlcCer 18:1;O2/16:0.
- **Acyl-linkages** (*N*- and/or *O*-) are annotated by FA C-atoms:DBE inside a separate pair of parentheses with proven position in front, e.g., Cer 18:1;O2/26:0;26O(FA 18:2).
- **Alkyl-linkages** (*N*- and/or *O*-) are annotated by C-atoms:DBE inside a separate pair of parentheses with proven position in front, e.g., FA 18:1(12Z);9O(16:1) for an ether lipid.
- When functional groups are part of lipid class abbreviation, e.g., PIP2 or SPBP, their proven positions are shown inside parentheses, separated by a comma if more than one, e.g., PIP2(4′,5′) 38:4 or SPBP (1) 18:1;O2.
- Greek letters are transcribed to Latin letters as follows: α to a, β to b, γ to g, δ to d, ω to w.
- Proven stereochemistry is shown after the respective functional group/side chain in square brackets [R] or [S], e.g., FA 20:4(6Z,8E,10E,14Z);5OH[S],12OH[R] = LTB_4_.

### Annotation of isotope-labeled lipids

- Isotope-containing lipid structures are indicated in square brackets annotating the isotope, followed by the number of isotopic atoms, e.g., FA 18:1[13C5].• Multiple isotopes are separated by a comma, e.g., FA 18:1[13C5,D4].
- When positions of isotopes are known, they are indicated in a separate pair of parentheses in front of the isotope number, e.g., FA 18:1[(14,15,16,17,18)13C5].
- Isotopes in fatty acyls or alkyls and in sphingoid bases are indicated in square brackets after the number of DBE, e.g., PC 34:1[D9] or PC O-16:0_18:1[13C5] and in Cer 34:1;O2[13C3], respectively. Isotopes in head groups of these structures are indicated in square brackets after class shorthand abbreviation, e.g., PC[D9] 34:1, TG[13C3] 54:3, SM[D9] 34:1;O2.
- When positions of isotopes in the lipid are not known, they are indicated in square brackets in front of class shorthand abbreviation, e.g., [D5]PC 34:1, [13C7]TG 54:3.

## FATTY ACYLS (FA)

### Fatty acyls

Shorthand abbreviations for Fatty Acyl classes are stated in **Table 2A**.

**TABLE 2A:** Class abbreviations in Category FA

Common Name	Lipid Class, LIPID MAPS	Abbreviation
Fatty acids	Fatty acids and conjugates [FA01]	FA
Fatty alcohols	Fatty alcohols [FA05]	FOH
Fatty aldehydes	Fatty aldehydes [FA06]	FAL
Acyl carnitines	Fatty acyl carnitines [FA0707]	CAR
Acyl CoAs	Fatty acyl CoAs [FA0705]	CoA
*N*-acyl amines	*N*-acyl amines [FA0802]	NA
*N*-acyl ethanolamines	*N*-acyl ethanolamines (endocannabinoids) [FA0804]	NAE
*N*-acyl taurines	*N*-acyl amines [FA0802]	NAT
Wax esters	Wax monoesters [FA0701]	WE
Wax diesters	Wax diesters [FA0702]	WD
FA estolides	FAHFA wax monoesters [FA0701]	FA-EST

Table 2B shows that lowest resolution level is based on *m/z* values, i.e., annotation at Species level (low mass resolution MS, e.g., carboxylate anion and oxygen atoms from functional groups). In addition, it is assumed that only a straight-chain fatty acid with or without DBE(s) is present. High mass resolution with accurate mass measurements may identify additional elements such as oxygen atoms of functional groups. Thus, a limited amount of structural information is provided at this level of analysis following the rules alluded to in the Annotation of lipid structures section, i.e., Species level. Annotation at DB-position level requires techniques such as ozonolysis (8) or photochemical derivatization (9) or GC-MS. The use of trivial or common names for even *simple* fatty acids implies that additional methods have been used to define the exact structure, such as a straight-chain, positions of DBs, or DB geometries. Chiral chromatography preceding MS/MS is required for respective stereochemistry. Because this is generally not routinely done, investigators should note in their reports when using a common name for a fatty acid that “The identity and stereochemistry of the fatty acid species reported using a common name (e.g., oleic acid, linolenic acid, arachidonic acid, etc.) is assumed based on biological intelligence”. This comment applies to *simple* as well as *complex *lipids that include fatty acids as part of the structure (e.g., glycerophospholipids, triacylglycerols, etc.). Examples for shorthand notation of fatty acids are presented in Table 2B.

**TABLE 2B:** Level-dependent shorthand notation for examples of fatty acids

Subclass	Species Level*^a^*^,^*^b^*	DB-Position Level*^c^*	Full Structure Level*^d^*	Complete Structure Level (= Common Name)*^e^*
Straight-chain FA	FA 12:0			Laurate
	FA 14:0			Myristate
	FA 16:0			Palmitate
	FA 16:1	FA 16:1(9)	FA 16:1(9Z)	Palmitoleate
	FA 18:0			Stearate
	FA 18:1	FA 18:1(9)	FA 18:1(9Z)	Oleate
	FA 18:1	FA 18:1(11)	FA 18:1(11E)	*tran*s-Vaccenate
	FA 18:2	FA 18:2(9, 12)	FA 18:2(9Z,12Z)	Linoleate
	FA 18:3	FA 18:3(9, 12, 15)	FA 18:3(9Z,12Z,15Z)	α-Linolenate
	FA 18:3	FA 18:3(6, 9, 12)	FA 18:3(6Z,9Z,12Z)	γ-Linolenate
	FA 18:4	FA 18:4(6, 9, 12, 15)	FA 18:4(6Z,9Z,12Z,15Z)	Stearidonate
	FA 20:0			Arachidate
	FA 20:3	FA 20:3(8, 11, 14)	FA 20:3(8Z,11Z,14Z)	*dihomo*-γ-Linolenate
	FA 20:3	FA 20:3(11, 14, 17)	FA 20:3(11Z,14Z,17Z)	
	FA 20:3	FA 20:3(5, 8, 11)	FA 20:3(5Z,8Z,11Z)	Mead acid
	FA 20:4	FA 20:4(5, 8, 11, 14)	FA 20:4(5Z,8Z,11Z,14Z)	Arachidonate
	FA 20:5	FA 20:5(5, 8, 11, 14, 17)	FA 20:5(5Z,8Z,11Z,14Z,17Z)	Eicosapentaenoate
	FA 22:0			Behenate
	FA 22:6	FA 22:6(4, 7, 10, 13, 16, 19)	FA 22:6(4Z,7Z,10Z,13Z,16Z,19Z)	Docosahexaenoate
	FA 24:0			Lignocerate
	FA 24:1	FA 24:1(15)	FA 24:1(15Z)	Nervonate
	FA 32:5	FA 32:5(14, 17, 20, 23, 26)	FA 32:5(14Z,17Z,20Z,23Z,26Z)	Dotriacontapentaenoic acid; FA 32:5(n-6)
	FA 34:5	FA 34:5 (19, 22, 25, 28, 31)	FA 34:5(19Z,22Z,25Z,28Z,31Z)	Tetratriacontapentaenoic acid; FA 34:5(n-3)
	FA 36:6	FA 36:6(18, 21, 24, 27, 30, 33)	FA 36:6(18Z,21Z,24Z,27Z,30Z,33Z)	Hexatriacontahexaenoic acid; FA 36:6(n-3)
Fatty acyl ester	FA 19:0		FA 18:0;1OMe	Methyl stearate
Methyl branched	FA 20:0		FA 16:0;3Me,7Me,11Me,15Me	FA 16:0;3Me,7Me[R],11Me[R],15Me (Phytanate)
Hydroxy	FA 18:0;O		FA 18:0;9OH	FA 18:0;9OH[S]
Oxo	FA 11:1;O*^b^*		FA 11:0;9oxo	FA 11:0;9oxo
Cyclopropane	FA 19:1		FA 19:0;[11-13cy3:0]	Lactobacillic acid
Cyclopropene	FA 19:2		FA 19:0;[9-11cy3:1(9)]	Sterculic acid
Cyclopentene	FA 18:3		FA 18:1(6Z);[14-18cy5:1(15)]	Gorlic acid

a Uncharged molecular mass measured by low resolution MS of corresponding *m/z* from carboxylate anion (electrospray ionization) or molecular ion species (radical cation by EI).

b Annotation based on the assumption of a straight-chain fatty acyl plus functional groups based on exact mass measurements using a high-resolution mass spectrometer of fatty acyl indicating ion.

c Positions of DBs determined by independent techniques such as ozonolysis (8) or photochemical derivatization (9).

d Shorthand notation applies only when exact location and nature of functional group(s) are determined by specific fragment ions obtained by derivatization and GC/MS or specific product ions in a MS/MS experiment.

e Validated assay is required to employ trivial names that engages appropriate internal standard, proper assessment of signal-to-noise, and a chromatographic based separation of potential isomers (GC or HPLC).

Fatty acyl esters, i.e., wax esters (WEs), wax diesters (WDs), fatty acyl estolides (FAHFAs, FA-EST), as well as *N*-acyl amines (NAs) and *N*-acyl ethanolamines (NAEs) are shown in Table 2C.

**TABLE 2C:** Level-dependent shorthand notation for examples of fatty aldehydes, esters, and amides

Subclass	Species Level	Molecular Species Level	DB-Position Level*^a^*	Full Structure Level*^b^*	Complete Structure Level (= Common Name)*^c^*
Fatty aldehyde	FAL 9:1;O	FAL 9:1;O	FAL 9:1(2);OH	FAL 9:1(2E);4OH	4-Hydroxynonenal
Wax ester*^d^*	WE 32:1	WE 14:0/18:1	WE 14:0/18:1(9)	WE 14:0/18:1(9Z)	WE 14:0/18:1(9Z)
Alkyl acetates*^d^*	WE 20:3	WE 18:3/2:0	WE 18:3(9,12,15)/2:0	WE 18:3(9Z,12Z,15Z)/2:0	WE 18:3(9Z,12Z,15Z)/2:0
Wax diester*^d^*	WD 42:0	WD 22:0/FA 10:0_FA 10:0	WD 22:0/FA 10:0_FA 10:0	WD 22:0;2O(FA 10:0),3O(FA 10:0)	WD 22:0;2O(FA 10:0[S]),3O(FA 10:0[R])
*N*-acyl amines (NA)*^d^*	NA 24:4	NA 4:0/20:4	NA 4:0/20:4(5,8,11,14)	NA 4:0/20:4(5Z,8Z,11Z,14Z)	NA 4:0/20:4(5Z,8Z,11Z,14Z)
*N*-acyl ethanolamines (NAE)*^d^*	NAE 18:2	NAE 18:2	NAE 18:2(9,12)	NAE 18:2(9Z,12Z)	NAE 18:2(9Z,12Z), anandamide 18:2(n-6)
Fatty acyl estolides (FA-EST)	FAHFA 36:1;O	FAHFA 18:1/18:0;O	FAHFA 18:1(9)/18:0;O	FAHFA 18:1(9Z)/9O(FA 18:0)	FAHFA 18:1(9Z)/9O(FA 18:0[R])

a Positions of DBs determined by independent techniques such as ozonolysis (8) or photochemical derivatization (9).

b Shorthand notation applies only when exact location and nature of functional group(s) are determined by specific fragment ions obtained by derivatization and GC/MS or specific product ions in a MS/MS experiment.

c Validated assay is required to employ trivial names that engages appropriate internal standard, proper assessment of signal-to-noise, and a chromatographic based separation of potential isomers (GC or HPLC).

d In shorthand notation for wax monoesters (WE), wax diesters (WD), and fatty amides (NA, NAE), alcohol and amine moieties precede the fatty acyl moiety.

### Oxygenated fatty acyls

Lipidomic studies of “oxygenated fatty acyls,” commonly referred to as “oxylipins” or “oxygenated PUFAs” in the literature, involves analysis of enzymatically and nonenzymatically generated lipids such as octadecanoids, eicosanoids, docosanoids, do- and tetratriacontanoids (Table 2D–F). Enzymatically generated isomers include prostaglandins, leukotrienes, and the various “specialized pro-resolving mediators,” i.e., lipoxins, protectins, maresins, and resolvin D/Es (Table 2F) (16). Nonenzymatic oxygenation of polyunsaturated fatty acids leads to numerous cyclic structures with various stereochemistry, such as phytoprostanes, isoprostanes, neuroprostanes, and all families of furans. Some of these isoprostanoids were identified over 25 years ago, particularly those of mammalian origin (17) and more recently also as components in foods of plant origin (18). The nomenclature for isoprostanoids is based on Taber, Morrow, and Roberts (19) and Rokach et al. (20), an update appeared in 2010 (21). Table 2G presents the precursor-product relationships for the classes of phytoprostanes, isoprostanes, and neuroprostanes, for which abbreviations PhytoP, IsoP, and NeuroP, respectively, have been proposed.

**TABLE 2D:** Shorthand notations for acyclic oxylipins at appropriate levels of annotation in lipidomic studies

Species Level*^a^*^,^*^b^*	DB-Position Level*^c^*	Structure Defined Level	Full Structure Level*^d^*	Complete Structure Level (= Common Name)*^e^*,*^f^*
FA 18:2;O	FA 18:2(9,11);O	FA 18:2;OH	FA 18:2(9Z,11E);13OH	13R-HODE, 13S-HODE
FA 20:4;O	FA 20:4(6,8,11,14);O	FA 20:4;OH	FA 20:4(6E,8Z,11Z,14Z);5OH	5R-HETE, 5S-HETE
FA 20:4;O	FA 20:4(5,8,10,14);O	FA 20:4;OH	FA 20:4(5Z,8Z,10E,14Z);12OH	12R-HETE, 12S-HETE
FA 20:4;O	FA 20:4(5,8,11,13);O	FA 20:4;OH	FA 20:4 (5Z,8Z,11Z,13E);15OH	15R-HETE, 15S-HETE
FA 20:4;O2	FA 20:4(6,8,10,14);O2	FA 20:4;(OH)2	FA 20:4(6Z,8E,10E,14Z);5OH,12OH	LTB_4_ (5S,12R)
FA 20:5;O3	FA 20:5(6,8,11,14,16);O3	FA 20:5;OOH;OH	FA 20:5(6E,8Z,11Z,14Z,16E);5OOH;18OH	5S-Hp-18S-HEPE
FA 20:5;O3	FA 20:5(6,8,10,14,16);O3	FA 20:5;(OH)3	FA 20:5(6Z,8E,10E,14Z,16E);5OH,12OH,18OH	Resolvin E1 (5S,12R,18R)
FA 22:6;O3	FA 22:6(4,8,10,12,14,19);O3	FA 22:6;(OH)3	FA 22:6(4Z,8E,10Z,12E,14E,19Z);7OH,16OH,17OH	Resolvin D2 (7S,16R,17S)
FA 22:6;O2	FA 22:6(4,8,10,12,16,19);O2	FA 22:6;(OH)2	FA 22:6(4Z,8E,10E,12E,16Z,19Z);7OH,14OH	Maresin 1 (7R,14S)

a Uncharged molecular mass measured by low resolution MS of corresponding *m/z* from carboxylate anion (electrospray ionization) or molecular ion species (radical cation by EI).

b Annotation based on the assumption of a straight-chain fatty acyl plus functional groups based on exact mass measurements using a high-resolution mass spectrometer of fatty acyl indicating ion.

c Positions of DBs determined by independent techniques such as ozonolysis (8) or photochemical derivatization (9).

d Shorthand notation applies only when exact location and nature of functional group(s) are determined by specific fragment ions obtained by derivatization and GC/MS or specific product ions in a MS/MS experiment.

e Common shorthand accepted by IUPAC (23).

f Validated assay is required to employ trivial names that engages appropriate internal standard, proper assessment of signal-to-noise, and a chromatographic based separation of potential isomers (GC or HPLC).

**TABLE 2E:** Shorthand notations for cyclic oxylipins at appropriate levels of annotation in lipidomic studies

Species Level*^a^*^,^*^b^*	Structure Defined Level	Full Structure Level*^c^*	Complete Structure Level (= Common Name)*^d^*,*^e^*
FA 20:4;O3	FA 20:3;(OH)2;oxo	FA 20:2(5Z,13E);[8-12cy5;11OH;9oxo];15OH	PGE_2 _
FA 20:4;O3	FA 20:3;(OH)2;oxo	FA 20:2(5Z,13E);[8-12cy5;9OH;11oxo];15OH	PGD_2_
FA 20:3;O3	FA 20:3;(OH)3	FA 20:2(5Z,13E);[8-12cy5;9OH,11OH];15OH	PGF_2α_
FA 20:3;O3	FA 20:2;(OH)2;oxo	FA 20:1(13E);[8-12cy5;11OH;9oxo];15OH	8-iso-PGE_1_
FA 20:3;O4	FA 20:2;(OH)3;oxo	FA 20:1(13E);[8-12cy5;9OH,11OH];15OH;6oxo	6-oxo-PGF_1α_
FA 20:3;O4	FA 20:3;(OH)3;oxy	FA 20:2(5Z,13E);[8-13cy6;9OH,11OH);11oxy];15OH	TXB_2 _
FA 22:5;O3	FA 22:5;(OH)3	FA 22:4(4Z,7Z,10Z,18E);[13-17cy5;14OH,16OH];20OH	20-F4-NeuroP

a Uncharged molecular mass measured by low resolution MS of corresponding *m/z* from carboxylate anion (electrospray ionization) or molecular ion species (radical cation by EI).

b Annotation based on the assumption of a straight-chain fatty acyl plus functional groups based on exact mass measurements using a high-resolution mass spectrometer of fatty acyl indicating ion.

c Shorthand notation applies only when exact location and nature of functional group(s) are determined by specific fragment ions obtained by derivatization and GC/MS or specific product ions in a MS/MS experiment.

d Common shorthand accepted by IUPAC (23).

e Validated assay is required to employ trivial names that engages appropriate internal standard, proper assessment of signal-to-noise, and a chromatographic based separation of potential isomers (GC or HPLC).

**TABLE 2F:** Parent polyunsaturated fatty acids and oxygenated product specialized pro-resolving mediators

Fatty Acid	Product Class	Complete Structure Level (= Common Name)
Arachidonic acid; AA(n-6)	Eicosanoid	Lipoxin A4, lipoxin B4
Eicosapentaenoic acid; EPA(n-3)	Eicosanoid	Resolvin E1, E2, E3
Docosahexaenoic acid; DHA(n-3)	Docosanoid	Resolvin D1, D2, D3, D4, D5, D6
Docosapentaenoic acid; DPA(n-3)	Docosanoid	Resolvin T1, T2, T3, T4
Docosahexaenoic acid; DHA(n-3)	Docosanoid	PCTR1, PCTR2, PCTR3, protectin D1/neuroprotectin D1
Docosahexaenoic acid; DHA(n-3)	Docosanoid	MCTR1, 2, 3, maresins 1, 2
Docosahexaenoic acid; DHA(n-3)	Docosanoid	Protectin DX
Dotriacontahexaenoic acid; FA 32:6(n-3)	Dotriacontanoid	Elovanoid ELV-N32
Tetratriacontahexaenoic acid; FA 34:6(n-3)	Tetratriacontanoid	Elovanoid ELV-N34

**TABLE 2G:** Parent polyunsaturated fatty acids and oxygenated product isoprostanoids

Fatty Acid	Product Class	Complete Structure Level (= Common Name)
α-Linoleic acid; ALA(n-3)	Octadecanoid	F1-PhytoP
γ-Linolenic acid; GLA(n-6)	Octadecanoid	F1-PhytoP_GLA_
Arachidonic acid; AA(n-6)	Eicosanoid	F2-IsoP
Eicosapentaenoic acid; EPA(n-3)	Eicosanoid	F3-IsoP
Adrenic acid; AdA(n-6)	Docosanoid	F2-IsoP_AdA_
Docosapentaenoic acid; DPA(n-6)	Docosanoid	F3-NeuroP_DPA(n-6)_
Docosapentaenoic acid; DPA(n-3)	Docosanoid	F3-NeuroP_DPA(n-3)_
Docosahexaenoic acid; DHA(n-3)	Docosanoid	F4-NeuroP
		FA 22:4(4Z,7Z,10Z,18E);[13-17cy5;14OH,16OH];20OH

Standards for structural validation by MS-inspection of these oxygenated fatty acids are described by Galano et al. (17) and are in agreement with those referred to for oxylipins (22). Specific shorthand nomenclature has been previously suggested and widely used for polyunsaturated oxygenated fatty acids (23).

The use of a common name (Table 2B, D, E) for fatty acyls or in reporting lipidomic studies also requires a high level of validation, typically with a representative biological sample using, for example, stable isotope dilution and chiral LC-MS/MS or capillary GC/MS with highly reproducible retention times for authentic standards. Otherwise, assumptions made on the basis of biological intelligence must be clearly stated.

## GLYCEROLIPIDS (GL)

See **Table 3A** and B for class abbreviations and examples, respectively. Lipid class abbreviation followed by number of C-atoms:number of DBE, for oxygenated lipids C-atoms:DBE;O-atoms, are as described in the Annotation of lipid structures section.

**TABLE 3A:** Class abbreviations in Category GL

Common Name	Lipid Class, LIPID MAPS	Abbreviation
Monoacyl/alkylglycerides (monoglycerides)	Monoradylglycerols [GL01]	MG
Diacyl/alkylglycerides (diglycerides)	Diradylglycerols [GL02]	DG
Triacyl/alkylglycerides (triglycerides)	Triradylglycerols [GL03]	TG
Estolides	Estolides [GL0305]	TG-EST
Sulfoquinovosylmonoacylglycerols	Glycosylmonoacylglycerols [GL0401]	SQMG
Monogalactosylmonoacylglycerol	Glycosylmonoacylglycerols [GL0401]	MGMG
Digalactosylmonoacylglycerol	Glycosylmonoacylglycerols [GL0401]	DGMG
Sulfoquinovosyldiacylglycerols	Glycosyldiacylglycerols [GL0501]	SQDG
Monogalactosyldiacylglycerol	Glycosyldiacylglycerols [GL0501]	MGDG
Digalactosyldiacylglycerol	Glycosyldiacylglycerols [GL0501]	DGDG

**TABLE 3B:** Examples for shorthand notation of glycerolipids

Bond Type	Species Level*^a^*	Molecular Species Level*^b^*	*sn*-Position Level*^c^*	Full Structure Level*^d^*
Acyl	MG 18:0	MG 18:0	MG 0:0/18:0/0:0	
Alkyl	MG O-18:0	MG O-18:0	MG 0:0/O-18:0/0:0	
Diacyl	DG 34:1	DG 16:0_18:1	DG 16:0/18:1/0:0	DG 16:0/18:1(9Z)/0:0
Acyl-alkyl	DG O-34:1	DG O-16:0_18:1	DG O-16:0/18:1/0:0	DG O-16:0/18:1(9Z)/0:0
Dialkyl	DG dO-32:1	DG O-16:0_O-16:1	DG O-16:0/O-16:1/0:0	DG O-16:0/O-16:1(9Z)/0:0
	DG 30:1*^e^*			
Triacyl	TG 52:2	TG 16:0_18:1_18:1	TG 16:0/18:1/18:1	TG 16:0/18:1(9Z)/18:1(11Z)
		TG 16:0_36:2 (only one acyl chain identified)	TG 16:0_18:1(sn-2)_18:1*^f^*	
Acyl-alkyl	TG O-52:2	TG O-16:0_18:1_18:1	TG O-16:0/18:1/18:1	TG O-16:0/18:1(9Z)/18:1(11Z)
	TG 51:2*^e^*			
Acyl-dialkyl	TG dO-52:2	TG O-18:1_O-16:0_18:1	TG O-18:1/O-16:0/18:1	TG O-18:1(9Z)/O-16:0/18:1(9Z)
	TG 50:2*^e^*			
Trialkyl	TG tO-52:2	TG O-18:1_O-16:0_O-18:1	TG O-18:1/O-16:0/O-18:1	TG O-18:1(9Z)/O-16:0/O-18:1(9Z)
	TG 49:2*^e^*			
TG-Estolide	TG 68:3;O2	TG 18:1_18:1_32:1;O2	TG 16:0;O(FA 16:0)/18:1/18:1	TG 16:0;5O(FA 16:0)/18:1(9Z)/18:1(9Z)

a Annotation based on exact mass measurements using a high-resolution mass spectrometer, which allows differentiation of isobaric acyl and alkyl species.

b Annotation requires MS/MS and detection of FA chain-specific fragments.

csn -Positions determined by specific analysis like differential mobility spectrometry (32), LC separation of isomeric species using silver ions (33).

d DB-positions determined by independent techniques such as ozonolysis (8) or photochemical derivatization (9).

e Annotation using low-resolution MS including the assumption of acyl chains only.

f Only acyl-chain at *sn*-2-position is defined.

Glycerolipids with known fatty acyl/alkyl constituents (molecular species):

- **separator** _: *sn*-position of acyl/alkyl constituents is **not known**. Constituents are presented in the order of increasing number of C-atoms, as are DB (DBE)-numbers for each C-atom number, e.g., TG 16:0_18:1_18:3.
- **separator /**: *sn*-position of acyl/alkyl constituents is **proven** (order *sn*-1/*sn*-2/*sn*-3; no FA linked 0:0), e.g., TG 16:0/18:3/18:1.
- When only one acyl chain of TG is known, it is presented in front of the sum of the remaining two acyl residues, e.g., TG 16:0_36:3.
- When only one of the *sn*-positions is defined, this is indicated inside a pair of parentheses, e.g., TG 16:0_18:1(sn-2)_18:0.

Other bond types than ester bonds are indicated as follows in front of the sum of C-atoms for acyl/alkyl constituents:

- O = alkyl, e.g., TG O-52:3
- *P* = proven O-alk-1-enyl-bond (acid-sensitive ether bond in “neutral plasmalogens” is *not* counted as a DB/DBE within the acyl-chain), e.g., TG P-52:3 or at higher resolution TG P-16:0/18:3/18:1.
- More than one “non”-ester bond is indicated in front of the bond type as d for *di*, t for *tri*, and e for *tetra*.

## GLYCEROPHOSPHOLIPIDS (GP)

See **Table 4A–C** for abbreviations and examples. Shorthand notation for phospholipid species contains abbreviation for phospholipid classes, followed by number of C-atoms:number of DBE, i.e., PS 36:4, for oxygenated lipids C-atoms:DBE;O-atoms, i.e., PS 36:3;O, as described in the Annotation of lipid structures section.

**TABLE 4A:** Class abbreviations in Category GP

Common Name	Lipid Class, LIPID MAPS	Abbreviation
Bis[monoacylglycero]phosphates	Monoacylglycerophosphomonoradylglycerols [GP0410]	BMP
Cardiolipins	Glycerophosphoglycerophosphoglycerols [GP12]	CL
Phosphatidic acids	Glycerophosphates [GP10]	PA
Phosphatidylcholines	Glycerophosphocholines [GP01]	PC
Phosphatidylethanolamines	Glycerophosphoethanolamines [GP02]	PE
Phosphatidylgylcerols	Glycerophosphoglycerols [GP04]	PG
Phosphatidylgylcerolphosphates	Glycerophosphoglycerophosphates [GP05]	PGP
Phosphatidylinositols	Glycerophosphoinositols [GP06]	PI
Phosphatidylserines	Glycerophosphoserines [GP03]	PS
Lysophospholipids		Prefix L
Phosphatidylinositol-mannoside		PIM
Subclasses phosphatidylinositol phosphates		
Phosphatidylinositol-monophosphates	Glycerophosphoinositol monophosphates [GP07]	PIP
Phosphatidylinositol-3-phosphates	Glycerophosphoinositol monophosphates [GP07]	PIP(3′)
Phosphatidylinositol-4-phosphates	Glycerophosphoinositol monophosphates [GP07]	PIP(4′)
Phosphatidylinositol-5-phosphates	Glycerophosphoinositol monophosphates [GP07]	PIP(5′)
Phosphatidylinositol-bisphosphates	Glycerophosphoinositol bisphosphates [GP08]	PIP2
Phosphatidylinositol-3,4-bisphosphates	Glycerophosphoinositol bisphosphates [GP08]	PIP2(3′,4′)
Phosphatidylinositol-3,5-bisphosphates	Glycerophosphoinositol bisphosphates [GP08]	PIP2(3′,5′)
Phosphatidylinositol-4,5-bisphosphates	Glycerophosphoinositol bisphosphates [GP08]	PIP2(4′,5′)
Phosphatidylinositol-trisphosphates	Glycerophosphoinositol trisphosphates [GP09]	PIP3
*N*-modified phospholipids		
*N*-alkyl PS		PS-N(Alk)
*N*-acyl PS		PS-N(FA)
Phosphatidylserine-carboxyalkylpyrroles		PS-CAP
Phosphatidylserine-malondialdehydes		PS-MDA
*N*-alkyl PE		PE-N(Alk)
*N*-acyl PE		PE-N(FA)
Phosphatidylethanolamine-carboxyalkylpyrroles		PE-CAP
Phosphatidylethanolamine-glucosides		PE-Glc
Phosphatidylethanolamine-glucuronides		PE-GlcA
Phosphatidylethanolamine-α-ketoglucoside		PE-GlcK
Phosphatidylethanolamine-carboxymethylates		PE-CM
Phosphatidylethanolamine-carboxyethylates		PE-CE
Phosphatidylethanolamine-formamides		PE-FA
Phosphatidylethanolamine-carbamides		PE-CA
Phosphatidyethanolamine- malondialdehydes		PE-MDA
Phosphatidylethanolamine-hydroxynonenals		PE-HNE
Phosphatidylethanolamine-isolevuglandins		PE-isoLG

**TABLE 4B:** Examples for shorthand notation of phospho- and lysophospholipids containing ester and/or ether bonds

Bond Type	Species Level*^a^*	Molecular SPECIES Level*^b^*	*sn*-Position Level*^c^*	Full Structure Level*^d^*
Diacyl	BMP 34:1	BMP 16:0_18:1	BMP 16:0/0:0/18:1/0:0 *sn*-2/*sn*-3/*sn*-2′/*sn*-3′	BMP 16:0/0:0/18:1(9Z)/0:0 *sn*-2/*sn*-3/*sn*-2′/*sn*-3′
Tetraacyl	CL 72:7	CL 18:1_18:2_18:2_18:2	CL 18:1/18:2/18:2/18:2 *sn*-1/*sn*-2/*sn*-1′/*sn*-2′	CL 18:1(9Z)/18:2(9Z,12Z)/18:2(9Z,12Z)18:2(9Z,12Z) *sn*-1/*sn*-2/*sn*-1′/*sn*-2′
		CL 18:1_54:6 (only one acyl chain identified)		
		CL 36:3_36:4 (known DG fragments)		
Tetra-alkyl	CL eO-80:0	CL O-20:0/O-20:0/O-20:0/O-20:0	CL O-20:0/O-20:0/O-20:0/O-20:0	CL O-16:0(3Me,7Me,11Me,15Me)/O-16:0(3Me,7Me,11Me,15Me)/O-16:0(3Me,7Me,11Me,15Me)/O-16:0(3Me,7Me,11Me,15Me)
Diacyl	PC 34:1*^e^*	PC 16:0_18:1	PC 16:0/18:1	PC 16:0/18:1(9Z)
Alkyl	PC O-34:1*^e^*	PC O-16:0_18:1	PC O-16:0/18:1	PC O-16:0/18:1(9Z)
Dialkyl	PC dO-34:1	PC O-16:0_O-18:1	PC O-16:0/O-18:1	PC O-16:0/O-18:1(9Z)
Diacyl	PE 34:1*^f^*	PE 16:0_18:1	PE 16:0/18:1	PE 16:0/18:1(9Z)
Plasmalogen	PE O-34:2*^f^*		PE P-16:0/18:1*^g^*	PE P-16:0/18:1(9Z)
Triacyl	LCL 54:5	LCL 18:1_18:2_18:2	LCL 18:1/18:2/18:2/0:0	LCL 18:1(9Z)/18:2(9Z,12Z)/18:2(9Z,12Z)/0:0
Monoacyl	LPC 16:0*^e^*	LPC 16:0	LPC 16:0/0:0	LPC 16:0/0:0
Monoalkyl	LPC O-16:0*^e^*	LPC O-16:0	LPC O-16:0/0:0	LPC O-16:0/0:0

a Annotation based on exact mass measurements using a high-resolution mass spectrometer, which allows differentiation of isobaric acyl and alkyl species.

b Annotation requires MS/MS and detection of FA chain specific fragments.

c sn-Positions determined by specific MS analysis like differential mobility spectrometry (34).

d Positions of DBs determined by independent techniques such as ozonolysis (8) or photochemical derivatization (9).

e Annotation using low resolution MS, QQQ and +PIS *m/z* 184 requires the assumption of even numbered carbon chains only.

f Annotation using low resolution MS, QQQ and +NL 141 requires the assumption of even numbered carbon chains only.

g Identification of plasmalogens (alk-1-enyl bond) require specific MS analysis (35).

**TABLE 4C:** Examples for shorthand notation of phosphatidylinositol phosphates

Bond Type	Species Level	Phosphate Position Level	Molecular Species Level	*sn*-Position Level	Full Structure Level
Diacyl	PIP 36:1	PIP(3′) 36:1	PIP(3′) 16:0_18:1	PIP(3′) 16:0/18:1	PIP(3′) 16:0/18:1(9Z)
Diacyl	PIP2 38:4	PIP2(4′,5′) 38:4	PIP2(4′,5′) 18:0_20:4	PIP2(4′,5′) 18:0/20:4	PIP2(4′,5′) 18:0/20:4(4Z,8Z,11Z,14Z)

### Phospholipids (PLs) and Lysophospholipids (LPLs)

Molecular species of phospholipids with known fatty acyl/alkyl constituents (Table 4B):

- **separator** _: *sn*-position of acyl/alkyl constituents is **not known**. Order of constituent presentation as described for glycerolipids, e.g., PC 16:0_18:2.
- **separator /**: *sn*-position of acyl/alkyl constituents is **proven** (*sn*-1/*sn*-2 or *sn*-2/*sn*-3); no constituent 0:0; e.g., PC 16:0/18:2.
- For BMP and CL classes *sn*-position order will be *sn*-2/*sn*-3/*sn*-2′/*sn*-3′ and *sn*-1/*sn*-2/*sn*-1′/*sn*-2′, respectively.
- When only one acyl chain or DG moieties of CL are known, sum of acyl residues are presented, e.g., CL 16:0_54:3 and CL 34:1_36:2, respectively.

Lysophospholipid classes are abbreviated as stated in LIPID MAPS nomenclature (Table 4A). Molecular species with unknown *sn*-position are presented as, e.g., LPE 18:1, with known *sn-*position as LPE 18:1/0:0 (Table 4B).

Other bond types than ester bonds are indicated as described for Glycerolipids, e.g., for an ether phospholipid PE O-18:0/18:2, for a “plasmalogen” PE P-18:0/20:4.

### Phosphatidylinositol phosphates (PIPs)

It is described in the Annotation of lipid structures section, when functional groups are part of lipid class abbreviation, their proven positions are shown directly at the abbreviation’s end inside parentheses, separated by a comma if more than one. A prominent example is PIP3(3′,4′,5′). Table 4C shows that “Phosphate position level” identifies phosphate position at inositol ring, i.e., PIP(3′) 38:4, otherwise it would be PIP 38:4. For ease of handling by databases, numbers of phosphates are not written in lower case.

### *N*-modified phospholipids and lysophospholipids

The amino function in PSs and PEs, including their lysoforms, is prone to react with a variety of electrophiles as has been shown in recent years (24). The products are generally termed *N*-mod PL and *N*-mod LPL in abbreviated form, common names and respective abbreviations are shown in Table 4A. Structures at Species-, Molecular species-, and *sn-*Position levels are presented in shorthand notation as described in the Annotation of lipid structures, Glycerolipids (GL), and Glycerophospholipids (GP) sections; specific examples are shown in Table 4D.

**TABLE 4D:** Examples for shorthand notation of *N*-modified phospholipids

Oxidative Modification	Species Level *^a^*	Molecular Species Level *^b^*	*sn*-Position Level *^c^*	Full Structure Level
*N*-alkyl	PS-N(Alk) 40:3	PS-N(6:0)16:0_18:3	PS-N(6:0) 16:0/18:3	PS-N(6:0) 16:0/18:3(9Z,12Z,15Z)
*N*-acyl	PE-N(FA) 54:5	PE-N(FA 18:1) 16:0_20:4	PE-N(FA 18:1) 16:0/20:4	PE-N(FA 18:1(9Z)) 16:0/20:4(4Z,8Z,11Z,14Z)
Hydroxynonenal adduct	PE-HNE 36:4	PE-HNE 16:0_20:4	PE-HNE 16:0/20:4	PE-HNE 16:0/20:4(4Z,8Z,11Z,14Z)

a Annotation based on exact mass measurements using a high-resolution mass spectrometer, which allows differentiation of isobaric acyl and alkyl species.

b Annotation requires MS/MS and detection of FA chain specific fragments.

c sn-Positions determined by specific MS analysis like differential mobility spectrometry (34).

### OxPLs

Phospholipids containing PUFA-constituents having methylene-interrupted *cis-*DBs (allylic DBs) and/or polar headgroups having amino-residues are susceptible to oxidation with formation of OxPLs. OxPL, so far, is a general term for a class of lipids produced by several processes that most often cannot be distinguished by MS analysis of the products. In all these cases, the products are called **OxPLs** (25).

Respective modes for production are the following:

- Oxygenation of PL to produce OxPL by direct action of lipoxygenases on PUFA constituents of PL gives rise to enzymatically produced specific oxPL. The stereochemistry of the resulting PUFA component usually reflects the specificity of the specific enzyme involved (26).
- The Land’s cycle is an alternative mechanism for enzymatic OxPL formation. Free, unesterified PUFAs liberated by phospholipase A_2_ and other enzymatic pathways from PL are first oxygenated by lipoxygenases, cyclooxygenases or CYP450 oxygenases. The resulting oxygenated PUFAs can then be reesterified into PLs resulting in the indirect enzymatic formation of specific oxPL.
- Nonenzymatic reactions are induced by free-radical oxygen/nitrogen species reacting directly with the PUFA constituents of PL or with free PUFAs which become incorporated into the PL by acyl transferases producing nonenzymatically derived oxPL. This oxygen transfer to PUFAs can further lead to DB rearrangement, cyclization and even truncation of such acyl-chains resulting in complex mixtures of oxPL (27).
- Nonradical reactive oxygen species like singlet oxygen or ozone can also contribute to PL oxidation with generation of full-chain or fragmented oxPL.
- PL having a polar head group with a modified amino-function (PE and PS) form a subclass named oxPL-Nmod.

Shorthand notation for **OxPLs** in general are presented in Table 4E.

**Table 4E:** Examples for shorthand notation of OxPLs

Oxidative Modification	Species Level*^a^*	Molecular Species Level*^b^*	*sn*-Position Level*^c^*	Structure Defined Level	Full Structure Level
Hydroxylation	PC 36:4;O	PC 16:0_20:4;O	PC 16:0/20:4;O	PC 16:0/20:4;OH	PC 16:0/20:4(5Z,8Z,10E,14Z);12OH
	PC 34:1;O2	PC 16:0_18:1;O2	PC 16:0/18:1;O2	PC 16:0/18:1;(OH)2	PC 16:0/18:1(9Z);12OH,13OH
Epoxide	PC 34:2;O	PC 16:0_18:2;O	PC 16:0/18:2;O	PC 16:0/18:1;Ep	PC 16:0/18:1(9Z);12Ep
Hydroperoxide	PC 34:2;O2	PC 16:0_18:2;O2	PC 16:0/18:2;O2	PC 16:0/18:2;OOH	PC 16:0/18:2(9Z,11E);13OOH
Peroxide	PC 34:2;O2	PC 16:0_18:2;O2	PC 16:0/18:2;O2	PC 16:0/18:1;OO	PC 16:0/18:1(9Z);12OO
Aldehyde	PC 21:1;O	PC 16:0_5:1;O	PC 16:0/5:1;O	PC 16:0/5:0;oxo	PC 16:0/5:0;5oxo
Carboxylic acid	PC 25:1;O2	PC 16:0_9:1;O2	PC 16:0/9:1;O2	PC 16:0/9:0;COOH	PC 16:0/9:0;8COOH
Hydroxy-aldehyde	PC 26:3;O2	PC 18:1_8:2;O2	PC 18:1/8:2;O2	PC 18:1/8:1;OH;oxo	PC 18:1(9Z)/8:1(6E);5OH;8oxo
PC *sn*-2 position	PC 36:4;O3	PC 16:0_20:4;O3	PC 16:0/20:4;O3	PC 16:0/20:2;[cy5;OH;oxo];OH	PC 16:0/20:2(5Z,13E);[8-12cy5;11OH;9oxo];15OH (common name 8-IsoPGE_2_-PC)

a Annotation based on exact mass measurements using a high-resolution mass spectrometer, which allows differentiation of isobaric acyl and alkyl species.

b Annotation requires MS/MS and detection of FA chain specific fragments.

c sn-Positions determined by specific MS analysis like differential mobility spectrometry (34).

## SPHINGOLIPIDS (SP)

Apart from sphingosine containing 18 C-atoms with two hydroxyl groups and one DB, other sphingoid bases reveal prominent backbones as well, particularly in brain or nonmammalian specimens (28). Consequently, the abbreviation SPB is strongly recommended as shorthand notation for the general term “sphingoid bases,” Cer for ceramides, and SM for sphingomyelins (**Table 5A**). Table 5B, C, and D define, in addition, shorthand notation according to structural resolution of sphingolipids. The updated rules for shorthand notation are the following:

- In case the long-chain base is not known, the sum composition of sphingoid base and fatty acid is shown as number of C-atoms:DBE;O-atoms, e.g., SPB 34:1;O2.
- In ceramides the sphingoid backbone is annotated C-atoms:DBE;O-atoms separated by a slash from the number of C-atoms:DBE;O-atoms of the *N*-linked fatty acid, e.g., Cer 18:1;O2/16:0.
- DB geometry and positions of hydroxyl groups (or other functional groups) are annotated as described for fatty-acyl-chains in Tab. 2B, e.g., Cer 18:1(4E);1OH,3OH/16:0.
- When the number of hydroxyl groups cannot be determined, numbers of C-atoms and DBE are assigned under the assumption of the number of hydroxyl groups in the major sphingoid base for that organism (e.g., dihydroxy in mammals).
- For further characterization of *N*-linked fatty acids, the rules as described in the Annotation of lipid structures section apply. The position of a fatty acid esterified to an *N*-linked hydroxy-fatty acyl is shown in a separate pair of parentheses xO(FA C-atoms:DBE) with x denoting the position of hydroxyl group (Δ nomenclature) in the *N*-linked fatty acids, e.g., Cer 18:1;O2/26:0;18O(FA 16:0).
- Any modification linked to a sphingoid base-OH is written in front of the (sub)class abbreviation with the integrated position number in parenthesis at the end of abbreviation, e.g., FA 24:1-ACer (1) 18:1;3OH/16:0 for an acylceramide, Gal-Cer (1) 18:0;3OH/16:0 for a galactosylceramide.
- Consequently, in shorthand notation from “Structure defined level” onwards only unmodified OH-groups of the sphingoid base are annotated.
- Shorthand notation for carbohydrate moieties is stated in Table 1C and examples are shown in Table 5D.
- For annotation of the sugar moiety in complex glycosphingolipids we refer to current practice in glycan science (https://www.ncbi.nlm.nih.gov/glycans) (15). When the sequence of sugars components is known, they are shown in this order separated by a hyphen. In case the sequence is unknown the components (followed by their number if more than one) are shown in alphabetic order in front of the respective lipid backbone. Annotation of the ceramide part follows the rules described above.
- Sphingoid base phosphates with unknown phosphate position are represented by SPBP, e.g., SPBP 18:1;(OH)2.
- Sphingoid base phosphates with known position of phosphate and of OH-positions is annotated by, e.g., SPBP (1) 18:1(4E);3OH.
- Ceramide phosphates with unknown phosphate position are represented by CerP, e.g., CerP 18:1;O2/16:0.
- Ceramide phosphates with known position of phosphate and of OH-positions are annotated by, e.g., CerP (1) 18:1(4E);3OH.
- Ceramide phosphates with 1,3 cyclic phosphate and known OH-positions are annotated by, e.g., CerP (1, 3) 18:1(4E).

**TABLE 5A:** Class abbreviations in Category SP

Common Name	Lipid Class, LIPID MAPS	Abbreviation
Sphingoid bases	Sphingoid bases [SP01]	SPB
Sphingoid base-phosphates	Sphingoid bases [SP0105]	SPBP
Ceramides	Ceramides [SP02]	Cer
Ceramide-phosphates	Ceramide phosphates [SP0205]	CerP
Acyl Ceramides	Acylceramides [SP0204]	ACer
Sphingomyelins	Phosphosphingolipids [SP03]	SM
Hexosylceramides	Neutral glycosphingolipids [SP05]	HexCer
Glucosylceramide	Neutral glycosphingolipids [SP05]	GlcCer
Galactosylceramide	Neutral glycosphingolipids [SP05]	GalCer
Dihexosylceramides	Neutral glycosphingolipids [SP05]	Hex2Cer
Lactosylceramide	Neutral glycosphingolipids [SP05]	LacCer
Sulfatides	Sulfoglycosphingolipids (sulfatides) [SP0602]	SHexCer
Inositolphosphorylceramides	Ceramide phosphoinositols [SP0303]	IPC (PI-Cer)
Ethanolaminephosphorylceramides	Ceramide phosphoethanolamines [SP0302]	EPC (PE-Cer)
Glycosylinositolphosphorylceramides	Ceramide phosphoinositols [SP0303]	GIPC
Mannosyl-inositolphosphoceramides	Ceramide phosphoinositols [SP0303]	MIPC
Mannosyl-diinositolphosphoceramide	Ceramide phosphoinositols [SP0303]	M(IP)2C

**TABLE 5B:** Examples for shorthand notation of sphingolipids with a free amino group

Sphingoid Base	Species Level*^a^*	Structure Defined Level	Full Structure Level*^b^*
Sphingosine	SPB 18:1;O2	SPB 18:1;(OH)2	SPB 18:1(4E);1OH,3OH
3-Keto-sphinganine	SPB 18:1;O2	SPB 18:0;OH;oxo	SPB 18:0;1OH;3oxo
Sphinganine	SPB 18:0;O2	SPB 18:0;(OH)2	SPB 18:0;1OH,3OH
Sphingadiene	SPB 18:2;O2	SPB 18:2;(OH)2	SPB 18:2(4E,14Z);1OH,3OH
Phytosphingosine	SPB 18:0;O3	SPB 18:0;(OH)3	SPB 18:0;1OH,3OH,4OH
C20-sphingosine	SPB 20:1;O2	SPB 20:1;(OH)2	SPB 20:1(4E);1OH,3OH
Sphingosine-1-phosphate	SPBP 18:1;O2	SPBP 18:1;OH	SPBP(1) 18:1(4E);3OH
Sphinganine-1-phosphate	SPBP 18:0;O2	SPBP 18:0;OH	SPBP(1) 18:0;3OH
1-Deoxymethyl-sphinganine	SPB 17:0;O	SPB 17:0;OH	SPB 17:0;2OH
1-Deoxy-sphinganine	SPB 18:0;O	SPB 18:0;OH	SPB 18:0;3OH
Lysoinositolphosphorylceramides	LIPC 18:0;O3	LIPC 18:0;(OH)2	LIPC(1) 18:0;3OH,4OH
Lysosphingomyelin	LSM 18:1;O2	LSM 18:1;OH	LSM(1) 18:1(4E);3OH

a Annotation based on exact mass measurements using a high-resolution mass spectrometer.

b Positions of functional groups and DBs determined by independent techniques such as chromatographic resolution, ozonolysis (8) or photochemical derivatization (9).

**TABLE 5C:** Examples for shorthand notation of sphingolipids containing an amide bound fatty acid

Phyla	Species Level*^a^*	Molecular Species Level*^b^*	Full Structure Level*^c^*
Mammalian	Cer 34:1;O2	Cer 18:1;O2/16:0	Cer 18:1(4E);1OH,3OH/16:0
Mammalian	Cer 34:0;O2	Cer 18:0;O2/16:0	Cer 18:0;1OH,3OH/16:0
Mammalian	ACer 58:1;O2	FA 24:1-ACer 18:1;O2/16:0	FA 24:1-ACer(1) 18:1(4E);3OH/16:0
Mammalian	CerP 34:1;O2	CerP 18:1;O2/16:0	CerP(1) 18:1(4E);3OH/16:0
Mammalian	SM 36:2;O2*^d^*	SM 18:2;O2/18:0	SM(1) 18:2(4E,14Z);3OH/18:0
Mammalian	SM 44:2;O2*^d^*	SM 20:1;O2/24:1	SM(1) 20:1(4E);3OH/24:1(15Z)
Mammalian	Cer 62:3;O4	Cer 18:1;O2/26:0;O(FA 18:1)*^e^*	Cer 18:1(4E);1OH,3OH/26:0;26O(FA 18:1(9Z))
		Cer 18:1;O2/44:2;O2*^f^*	
Plant	IPC 42:1;O4	IPC 18:1;O3/24:0;O	IPC(1) 18:1(8E);3OH,4OH/24:0;2OH
Yeast	Cer 44:0;O5	Cer 18:0;3O/26:0;O2	Cer 18:0;1OH,3OH,4OH/26:0;2OH,3OH

a Annotation based on exact mass measurements using a high-resolution mass spectrometer.

b Annotation requires MS/MS enabling detection of sphingoid base and/or *N*-linked FA.

c Positions of functional groups and DBs determined by independent techniques such as chromatographic resolution, ozonolysis (8) or photochemical derivatization (9).

d Annotation using low resolution MS QQQ and a PIS *m/z* 184 requires the assumption of a sphingoid base with two hydroxyl groups.

e Annotation with structural characterization of O-acyl in *N*-linked acyl chain.

f Annotation without structural differentiation of *N*-linked acyl chain.

**TABLE 5D:** Examples for shorthand notation of glycosphingolipids containing an amide bound fatty acid

Phyla	Species Level*^a^*	Molecular Species Level*^b^*	Full Structure Level*^c^*
Mammalian	Hex-Cer 34:1;O2	Hex-Cer 18:1;O2/16:0	Glc-Cer(1) 18:1(4E);3OH/16:0 (see also Fig. 1)
		Glc-Cer 18:1;O2/16:0 *^d^*	
Mammalian	Hex-Cer 34:0;O2	Hex-Cer 18:0;O2/16:0	Gal-Cer(1) 18:0;3OH/16:0
		Gal-Cer 18:0;O2/16:0*^d^*	
Mammalian	Hex2Cer 34:1;O2	Hex2Cer 18:1;O2/16:0	Lac-Cer(1) 18:1(4E);3OH/16:0*^e^*
			Gal-Glc-Cer(1) 18:1(4E);3OH/16:0
Mammalian	Hex3Cer 42:1;O2	Hex3Cer 18:1;O2/24:0	Gal-Gal-Glc-Cer(1) 18:1(4E);3OH/24:0 (= Gb3)
Mammalian	NeuAcHex2Cer 42:1;O2	NeuAcHex2Cer 18:1;O2/24:0	NeuAc-Gal-Glc-Cer(1) 18:1(4E);3OH/24:0 (= GM3)
Mammalian	NeuAc2Hex2Cer 42:1;O2	NeuAc2Hex2Cer 18:1;O2/24:0	NeuAc-NeuAc-Gal-Glc-Cer(1) 18:1(4E);3OH/24:0 (= GD3)
Mammalian	SHex-Cer 34:1;O2	SHex-Cer 18:1;O2/16:0	S(3′)Hex-Cer(1) 18:1(4E);3OH/16:0
			S(3′)Gal-Cer(1) 18:1(4E);3OH/16:0*^e^*
Mammalian	SHexHexNAcHex3Cer 34:1;O2	SHexHexNAcHex3Cer 18:1;O2/16:0	S(3′)Hex-HexNac-Hex-Hex-Hex-Cer(1) 18:1(4E);3OH/16:0
			S(3′)Gal-GalNAc-Gal-Gal-Glc-Cer(1) 18:1(4E);3OH/16:0*^e^* (globopentaosylceramide sulfate)
Plant	HexA-IPC 42:1;O4	HexA-IPC 18:1;O3/24:0;O	GlcA-IPC(1) 18:1(8E);3OH,4OH/24:0;2OH
Plant	HexHexA-IPC 42:1;O4	Hex-HexA-IPC 18:1;O3/24:0;O	Glc-GlcA-IPC(1) 18:1(8E);3OH,4OH/24:0;2OH
Plant	HexAHexNAc-IPC 42:1;O4	HexNAc-HexA-IPC 18:1;O3/24:0;O	GlcNAc-GlcA-IPC(1) 18:1(8E);3OH,4OH/24:0;2OH
Plant	HexHexAHexNAc-IPC 42:1;O4	Hex-HexNAc-HexA-IPC 18:1;O3/24:0;O	Glc-GlcNAc-GlcA-IPC(1) 18:1(8E);3OH,4OH/24:0;2OH
Yeast	MIPC 44:0;O4	MIPC 18:0;O3/26:0;O	MIPC(1) 18:0;3OH,4OH/26:0;2OH
Yeast	M(IP)2C 46:0;O4	M(IP)2C 20:0;O3/26:0;O	M(IP)2C(1) 20:0;3OH,4OH/26:0;2OH

a Annotation based on exact mass measurements using a high-resolution mass spectrometer.

b Annotation requires MS/MS enabling detection of sphingoid base and/or *N*-linked FA.

c Positions of functional groups and DBs determined by independent techniques such as chromatographic resolution, ozonolysis (8) or photochemical derivatization (9).

d Separation of isomeric hexosylceramide by HILIC (36).

e Annotation requires separation of stereoisomers at glycosidic linkage (α/β).

## STEROLS (ST)

We use the term sterol to embrace all molecules based on the cyclopentanoperhydrophenanthrene skeleton. **In the case of sterols, the ring system does not add to the number of DBE.** Endogenously biosynthesized mammalian sterols are derived from cholesterol or its precursors, yet plant and yeast sterols can also be a source via the food chain. The stereochemistry of the cholesterol molecule is maintained to a large extent by mammalian sterols, which all contain at least one hydroxyl or oxo group attached to carbon 3. High resolution MS with accurate mass may identify other functional groups, as will MS/MS or MS^n^ scans. Stereochemistry can often be defined by comparing the chromatographic retention time to authentic standards and, in some cases, by MS/MS or MS^n^. The class abbreviations within category ST are shown in **Table 6A**.

**TABLE 6A:** Class abbreviations in Category ST

Common Name	Lipid Class, LIPID MAPS	Abbreviation
Sterols	Sterols [ST01]	ST
Sterol esters	Sterol esters [ST0102]	SE
Bile acids	Bile acids and derivatives [ST04]	BA
Free cholesterol = cholesterol		FC
Cholesteryl ester	Cholesteryl esters [ST0102]	CE
Sterylglycosides	Sterylglycosides	SG
Acylsterylglycosides	Monoradylglycosterols	ASG

The following rules for shorthand nomenclature have been adopted in the examples given in Table 6B.

- In shorthand notation the category abbreviation ST is used as class abbreviation. In some cases, other abbreviations e.g., FC, CE, BA, SE, SG and ASG can be used. In all cases, class abbreviation is followed by number of carbon atoms:number of DB, and separated by semicolon is the number of oxygens, e.g., ST 27:1;O for cholesterol and lathosterol (also zymostenol), or ST 24:1;O5 for an oxidized sterol *and* for cholic acid *and* ursocholic acid. The latter is an important point: Some bile acids have an identical mass and molecular formula to oxidized sterols lacking a carboxylic acid group. This must be considered, when class abbreviation “BA” is used.
- Shorthand notation of further functional groups are written, separated by a semicolon, after the number of oxygens, e.g., BA 24:1;O5;T for taurocholic acid (= common name, abbreviation TCA).
- Following the number of double bonds, proven position and stereochemistry is shown. R and S configurations are preferred for side-chain stereochemistry and are shown in square brackets. α (below ring/plane), written as a, and β (above ring/plane), written as b, are preferred for ring stereochemistry, e.g. 3aOH and 17bOH. Stereochemistry at C-5 introduced by reduction of the Δ^5^ bond is indicated by 5aH or 5bH. Replacing the number of oxygens, proven positions and stereochemistry of oxygen containing functional groups are shown. If such stereochemistry is known the common name of the compound can be used.
- The side-chain at carbon-17 of the cyclopentanoperhydrophenanthrene skeleton always has b-stereochemistry (17b) and consequently is not presented in the shorthand annotation.
- For structures fully proven or based on assumption by biological intelligence, such as e.g., cholesterol, cholesteryl esters, steryl esters, bile acids, sterylglycosides, and acylsterylglycosides abbreviations FC, CE, SE, BA, SG and ASG, respectively, can be used as shown in Table 6A. CE is followed by number of C-atoms:number of DBE of the fatty acid esterified to the hydroxyl group at position 3, e.g., CE 18:2 (Table 6B). Shorthand notation SE is used as above followed by slash (for monohydroxysterols) or underscore (for polyhydroxysterols) number of C-atoms:number DBE of the fatty acid esterified to the hydroxyl group (Table 6B).
- MS/MS scans reveal the presence of conjugates: Taurine (T) and glycine (G) each are conjugated through an amide bond to the carboxylic acid group of bile acids, respective amide bonds with conjugates are designated in shorthand notation “COT” and “COG” (Table 6B); sulfuric acid (S) is conjugated to a hydroxyl group through an ester bond; glucuronic acid (GlcA), *N*-acetylglucosamine (GlcNAc), and hexose (Hex) sugars are assumed to be linked to a hydroxyl group through an acetal linkage (Table 6B).
- In the case full stereochemistry is known the common names as presented in Table 6B can be used.

**TABLE 6B:** Examples of shorthand notation for sterols

Lipid Class	Species Level	Full Structure Level	Complete Structure Level (= Common Name)
ST (FC)	ST 27:1;O	ST 27:1(5Z);3bOH = FC	Cholesterol
ST	ST 27:1;O	ST 27:1(7);5aH;3bOH	Lathosterol
ST	ST 28:3;O	ST 28:3(5Z,7Z,22E);24Me[R];3bOH	Ergosterol
ST	ST 27:2;O3	ST 27:1(5Z);3bOH;26COOH[25R]	3β-Hydroxycholest-5-en-(25R)26-oic acid
SE	SE 27:1/16:0	CE 16:0	Cholesteryl palmitate
SE	SE 27:1/18:2	CE 18:2(9Z,12Z)	Cholesteryl linoleate
SE	SE 27:2/18:1	SE 27:2(8E,24);5aH/18:1(9Z)	Zymosteryl oleate
ST	ST 21:3;O2	ST 21:1(4Z);3oxo,20oxo	Progesterone
ST	ST 19:2;O2	ST 19:1(4Z);17bOH;3oxo	Testosterone
ST	ST 19:2;O2	ST 19:1(5Z);3bOH;17oxo	Dehydroepiandrosterone
ST	ST 18:3;O2	ST 18:3(1,3,5);3OH,17bOH	17β-Estradiol
ST	ST 19:2;O2;S	ST 19:1(5Z);3bS;17oxo	Dehydroepiandrosterone sulfate
BA	ST 24:1;O5	BA 24:0;5bH;3aOH,7aOH,12aOH;24COOH	Cholic acid (CA)
BA	ST 24:1;O3	BA 24:0;5bH;3aOH;24COOH	Lithocholic acid (LCA)
BA	BA 24:1;O5;T	BA 24:0;5bH;3aOH,7aOH,12aOH;24COT	Taurocholic acid (TCA)
BA	BA 24:1;O4;G	BA 24:0;5bH;3aOH,7aOH;24COG	Glycochenodeoxycholic acid (GCDCA)
BA	ST 24:1;O4;HexNAc	BA 24:0;5bH;3aOH,7bOGlcNAc;24COOH	Ursodeoxycholic acid 7β-*N*-acetylglucosaminide (UDCA-GlcNac)
SG	SG 27:1;O;Hex	SG 27:1(5Z);3bOGlc	Cholesteryl glucoside
ASG	ASG 29:2;O;Glc;FA20:3	ASG 29:2(5Z,22E);24Et[S];3bOGlc;6O(FA 20:3)	20:3(11Z,14Z,17Z)-Glc-stigmasterol

## DISCUSSION AND CONCLUSIONS

This publication updates both the classification and nomenclature (2, 3) and shorthand notation (4), and targets two goals. First, to emphasize and enable correct reporting of mass spectrometric data according to the resolving power of MS instrument platforms operating in high-resolution (and often high-throughput) mode. Second, to provide a comprehensible shorthand notation for the lipids commonly analyzed. Such common nomenclature is essential for standardized reporting of lipid species data and construction of data resources. Moreover, standardized data facilitate automated datamining and import into databases by script-based algorithms with only minimal data curation. Related data repositories require a hierarchical concept mirroring the structural resolution provided by mass spectrometric analysis reflected in the presented shorthand notation. To this end, the LMSD database, respective MS search tool, and, in particular, shorthand notations for all relevant lipids are now available on the LMSD detail view pages at “Species level” and “Molecular species level”, the latter embracing “Phosphate-”, “DB-”, and “*sn*-position level”. In a few instances, however, easy use of this shorthand notation by lipidomics experts has priority over its stringent use in a bioinformatics format.

A standardized annotation for lipid species, as a common language, is a key component to promote and further advance this emerging omics discipline (29). Therefore, the Lipidomics Standards Initiative (LSI; https://lipidomics-standards-initiative.org/) has been recently introduced (13), pursuing development of guidelines and channeling community-wide efforts in close collaborations with LIPID MAPS (https://www.lipidmaps.org/) as has been emphasized recently (30). In addition, alignment with other initiatives, as for example, adaptation of mzTab-M, a data format developed for metabolomics (31), to the presented nomenclature is possible.

In summary, the shorthand nomenclature presented here is viewed as a standard in lipidomics that can be updated periodically.

### Data availability

All data are contained within this article.
